# Supplementary material for: Bidirectional Wnt signaling between endoderm and mesoderm confers tracheal identity in mouse and human cells
Source: Nat Commun. 2020 Aug 27;11:4159. doi: 10.1038/s41467-020-17969-w (PMC7453000; doi:10.1038/s41467-020-17969-w)
Supplement: Supplementary file 2 — Reporting Summary [file 41467_2020_17969_MOESM2_ESM.pdf]

nature research

Corresponding author(s): Mitsuru Morimoto

Last updated by author(s): Jul 22, 2020

Reporting Summary

Nature Research wishes to improve the reproducibility of the work that we publish. This form provides structure for consistency and transparency in reporting. For further information on Nature Research policies, see Authors & Referees and the Editorial Policy Checklist .

Statistics

For all statistical analyses, confirm that the following items are present in the figure legend, table legend, main text, or Methods section.

n/a	Confirmed	
☐	☒	The exact sample size (*n*) for each experimental group/condition, given as a discrete number and unit of measurement
☐	☒	A statement on whether measurements were taken from distinct samples or whether the same sample was measured repeatedly
☐	☒	The statistical test(s) used AND whether they are one- or two-sided<br>*Only common tests should be described solely by name; describe more complex techniques in the Methods section.*
☒	☐	A description of all covariates tested
☐	☒	A description of any assumptions or corrections, such as tests of normality and adjustment for multiple comparisons
☐	☒	A full description of the statistical parameters including central tendency (e.g. means) or other basic estimates (e.g. regression coefficient) AND variation (e.g. standard deviation) or associated estimates of uncertainty (e.g. confidence intervals)
☐	☒	For null hypothesis testing, the test statistic (e.g. *F*, *t*, *r*) with confidence intervals, effect sizes, degrees of freedom and *P* value noted<br>*Give P values as exact values whenever suitable.*
☒	☐	For Bayesian analysis, information on the choice of priors and Markov chain Monte Carlo settings
☒	☐	For hierarchical and complex designs, identification of the appropriate level for tests and full reporting of outcomes
☒	☐	Estimates of effect sizes (e.g. Cohen's *d*, Pearson's *r*), indicating how they were calculated

Our web collection on statistics for biologists contains articles on many of the points above.

Software and code

Policy information about availability of computer code

Data collection	Images were taken by Zeiss 710 confocal microscope (Carl Zeiss) or IX83 microscrope (OLYMPUS) or Nicon WIdefield microscope (Nicon)<br>qPCR analyses were performed by QuantStudio 3 and 6
Data analysis	Images were analyzed by Excel 2013(Microsoft) IMARIS 8.4.1 (Bitplane), Fiji v1.52, or ZEN 2011 SP3 (black) version 8.1.11.484 (Carl Zeiss) software.<br>Statistical analyses were done by GraphPad PRISM8 (GraphPad software)

For manuscripts utilizing custom algorithms or software that are central to the research but not yet described in published literature, software must be made available to editors/reviewers. We strongly encourage code deposition in a community repository (e.g. GitHub). See the Nature Research guidelines for submitting code & software for further information.

Data

Policy information about availability of data

All manuscripts must include a data availability statement. This statement should provide the following information, where applicable:

- Accession codes, unique identifiers, or web links for publicly available datasets
- A list of figures that have associated raw data
- A description of any restrictions on data availability

The authors declare that all data supporting the findings of this study are available within the article and its Supplementary Information files or from the corresponding author upon reasonable request.
The Source data underlying Figs. 4b-e, 4g, 4i, 5b-e, 5g, 5i and Supplementary Figs. 6 and 7 were provided as a Source data file.
The datasets generated during the current studies will be available before publication in the System Science of Biological Dynamics (SSBD) database (http://ssbd.qbic.riken.jp/set/20200722/). DOI is https://doi.org/10.24631/ssbd.repos.2020.07.003

Field-specific reporting

Please select the one below that is the best fit for your research. If you are not sure, read the appropriate sections before making your selection.

- [x] Life sciences
- [ ] Behavioural & social sciences
- [ ] Ecological, evolutionary & environmental sciences

For a reference copy of the document with all sections, see nature.com/documents/nr-reporting-summary-flat.pdf

Life sciences study design

All studies must disclose on these points even when the disclosure is negative.

Sample size

No sample size calculation was performed. Sample size was dicided by the standard in the field.
At least 3 embryos for mouse experiments and 3 wells for cell culture experiments were analyzed in each experiments.
This is based on the number of biological replicates required for statistical analyses.

Data exclusions

No data were excluded in this study.

Replication

The number of replicates was described in the legends of figures.
In Figure 1b, c, d, 3 embryos per genotype from more than two 2 littermate were analyzed.
In Figure 2a, b, d, e, 3 embryos per genotype were analyzed.
In Figure 2c , 2 embryos were analyzed.
In Figure 3a, c , e, 2 embryos per genotype were analyzed.
In Figure 3b, 3 embryos per genotype were analyzed.
In FIgure 4 and 5 for mESC and hESC experiments, data was collected from 3 wells per individual condition. And similar experiments were repeated at least twice. All attempts for replicate were successful.

Randomization

Randomization was not applicable in this study, samples were chosen based on genotype.

Blinding

Formal blinding was not performed in the study. This was technically difficult for us because sample collection and analyses were done by same researcher. We treated and analysed all samples in the similar procedure.

Reporting for specific materials, systems and methods

We require information from authors about some types of materials, experimental systems and methods used in many studies. Here, indicate whether each material, system or method listed is relevant to your study. If you are not sure if a list item applies to your research, read the appropriate section before selecting a response.

Materials & experimental systems

n/a	Involved in the study	
☐	☒	Antibodies
☐	☒	Eukaryotic cell lines
☒	☐	Palaeontology
☐	☒	Animals and other organisms
☒	☐	Human research participants
☒	☐	Clinical data

Methods

n/a	Involved in the study	
☒	☐	ChIP-seq
☒	☐	Flow cytometry
☒	☐	MRI-based neuroimaging

Antibodies

Antibodies used

We listed antibodyies in Supplementary Information.
Anti-Aggrecan, Abcam, ab3778
Anti-CDH1, Cell Signaling technology, #3195
Anti-Collagen 1a1, Abcam, ab34710
Anti-Collagen 2a1, Santa Cruz Biotechnology, Inc, sc-7764
Anti-DIG-AP, Roche, 11093274910
Anti-Foxf1, R&D systems, AF4798
Anti-Gata4, Santa Cruz Biotechnology, Inc, sc-1237
Anti-GFP, Thermo FIsher Scientific, A10262
Anti-Nkx2.1, Santa Cruz Biotechnology Inc, sc-13040
Anti-Nkx6.1, Developmental Studies Hybridoma Bank, F55A12
Anti-Nkx6.1, R&D systems, AF5857
Anti-SMA, Sigma, #A2547

Anti-SMA-Cy3, Sigma, C6198
Anti-Sox2, Santa Cruz Biotechnology, Inc, sc-17320
Anti-Sox9,Abcam, AB5535
Anti-Tagln, Abcam, ab14106
Anti-Tbx4, Abcam, ab220035

Validation

Anti-Aggrecan, Abcam, ab3778
Species and Application by Manufacturer;
A mouse monoclonal antibody recommended for detection of Mouse, Cow, Human Aggrecan by ELISA, IHC-P, IHC-Fr, WB, ICC/IF.
(https://www.abcam.com/aggrecan-antibody-6-b-4-ab3778.html)

Anti-CDH1, Cell Signaling technology, #3195
E-Cadherin (24E10) Rabbit mAb detects endogenous levels of total E-cadherin protein.
The antibody does not cross-react with related family members, such as N-cadherin.
Species Reactivity:
Human, Mouse
Species predicted to react based on 100% sequence homology:
Bovine, Dog, Pig
AApplication Dilution
Western Blotting 1:1000
Immunohistochemistry (Paraffin) 1:400
Immunohistochemistry (Frozen) 1:200
Immunofluorescence (Immunocytochemistry) 1:200
Flow Cytometry 1:200
(https://media.cellsignal.com/pdf/3195.pdf)

Anti-Collagen 1a1, Abcam, ab34710
Species and Application by Manufacturer;
A rabbit polyclonal antibody recommended for detection of Mouse, Rat, Sheep, Goat, Horse, Cow, Human, Pig, Common marmoset Collagen I by IHC-Fr, Indirect ELISA, WB, IHC-P, ELISA, ICC/IF, IP
(https://www.abcam.com/collagen-i-antibody-ab34710.html)

Anti-Collagen 2a1, Santa Cruz Biotechnology, Inc, sc-7764
Species and Application by Manufacturer;
A goat polyclonal antibody recommended for detection of Mouse, Rat, Human, Xenopus Collagen 2a1 by WB, ELISA, ICC/IF, IP
(http://datasheets.scbt.com/sc-7764.pdf)

Anti-DIG-AP, Roche, 11093274910
Species and Application by Manufacturer;
The polyclonal antibody from sheep is specific to digoxigenin and digoxin and shows no cross-reactivity with other steroids, such as human estrogens and androgens. Use Anti-Digoxigenin-AP, Fab fragments for the detection of digoxigenin-labeled compounds using:
• cDNA array
• Colony/plaque hybridization
• Dot blot
• ELISA
• Gel shift assay
• Immunohistocytochemistry
• In situ hybridization
• Nonradioactive DNA sequencing blot
• Northern blot
• RNase protection assay
• Southern blot
• Western blot
• Fluorescent in situ hybridization
• Section in situ hybridization and whole mount in situ hybridization
• Electrophoretic mobility shift assay
(https://www.sigmaaldrich.com/catalog/product/roche/11093274910?lang=en&region=US)
Anti-Foxf1, R&D systems, AF4798
Species and Application by Manufacturer;
A goat polyclonal antibody recommended for detection of Foxf1 of mouse and human by WB.
(https://www.rndsystems.com/products/human-mouse-foxf1-antibody_af4798)

Anti-Gata4, Santa Cruz Biotechnology, Inc, sc-1237
Species and Application by Manufacturer;

A goat polyclonal antibody recommended for detection of Mouse, Rat, Human Gata4 by WB, IP, IF
(http://datasheets.scbt.com/sc-1237.pdf)

Anti-GFP, Thermo FIsher Scientific, A10262
Species and Application by Manufacturer; Polyclonal antibody from Chick recognized GFP-tag. Tested applications are ICC, IF, WB.
(https://www.thermofisher.com/antibody/product/GFP-Antibody-Polyclonal/A10262)

Anti-Nkx2.1, Santa Cruz Biotechnology Inc, sc-13040
Species and Application by Manufacturer;
Rabbit polyclonal antibody recommended for detection of Nkx2.1 of Mouse, Rat and Human origin by WB, IP, IF, IHC(P) and ELISA; also reactive with additional species, including and Equine, Canine, Bovine and Porcine.
(https://www.scbt.com/scbt/product/ttf-1-antibody-h-190?productCanUrl=ttf-1-antibody-h-190&_requestid=1343)

Anti-Nkx6.1, Developmental Studies Hybridoma Bank, F55A12
Species and Application by Manufacturer;
A mouse monoclonal antibody recommended for detection of Mouse, Rat, Human, Bovine, Pig Nkx6.1 by FC/FACS, B, IF.
(https://www.citeab.com/antibodies/149953-f55a12-homeobox-protein-nkx-6-1-nkx6-1)

Anti-Nkx6.1, R&D systems, AF5857
Species and Application by Manufacturer;
A goat polyclonal antibody recommended for detection of Human and Mouse NKX6.1 in direct ELISAs and WB. In direct ELISAs, less than 1% cross-reactivity with recombinant Human NKX3.1 is observed.
(https://www.rndsystems.com/products/human-mouse-nkx61-antibody_af5857)

Anti-SMA, Sigma, #A2547
Species and Application by Manufacturer;
A mouse monoclonal antibody recommended for Human, Frog, Sheep, Chicken, Goat, Bovine, Rat, Guinea pig, Mouse, Canine, Rabbit, Snake SMA by IF, IHC, WB
(https://www.sigmaaldrich.com/catalog/product/sigma/a2547?lang=en&region=US)

Anti-SMA-Cy3, Sigma, C6198
Species and Application by Manufacturer;
The antibody (also known as anti-α-Sm-1) is specific for the single isoform of α-smooth muscle actin of rabbit, guinea pig, mouse, chicken, snake, sheep, goat, human, frog, rat, canine, bovine. It reacts specifically with α-smooth muscle actin in immunoblotting assays and labels smooth muscle cells in frozen or formalin-fixed, paraffin-embedded tissue sections. Applications in which this antibody has been used successfully were Flow cytometry/Cell sorting (2 papers)
Immunocytochemistry (3 papers)
Immunofluorescence (13 papers)
Immunohistochemistry (27 papers)
Western Blotting (2 papers)
(https://www.sigmaaldrich.com/catalog/product/sigma/c6198?lang=en&region=US)

Anti-Sox2, Santa Cruz Biotechnology, Inc, sc-17320
Species and Application by Manufacturer;
A goat polyclonal antibody recommended for detection of Sox-2 of mouse, rat, human and avian origin by WB, IP, IF, IHC(P) and ELISA; also reactive with additional species, including and equine, canine, bovine, porcine and avian.
(https://www.scbt.com/scbt/product/sox-2-antibody-y-17?productCanUrl=sox-2-antibody-y-17&_requestid=165)

Anti-Sox9,Abcam, AB5535
Species and Application by Manufacturer;
A rabbit polyclonal antibody recommended for detection of Sox-9 of mouse, rat, human and avian origin by WB, ChIP, IF, IHC, ChIP-Seq, and ICC
(http://www.emdmillipore.com/US/en/product/Anti-Sox9-Antibody,MM_NF-AB5535?bd=1)

Anti-Tagln, Abcam, ab14106
Species and Application by Manufacturer;
A rabbit polyclonal antibody recommended for detection of Mouse, Rat, Chicken, Cow, Human, Pig Tagln by WB, IHC, ICC, ICC/IF.
(https://www.abcam.com/taglntransgelin-antibody-ab14106.html)

Anti-Tbx4,Abcam, ab220035
Species and Application by Manufacturer;
A rabbit polyclonal antibody recommended for detection of Tbx4 of mouse, human and dog by IF, and ICC.
(https://www.abcam.com/tbx4-antibody-ab220035-protocols.html)

Eukaryotic cell lines

Policy information about cell lines

Cell line source(s)	Human ES cells, H1 cells (WiCell, NIH approval number NIHhESC-10-0043 and NIHhESC-10-0062), were provided by Pluripotent Stem Cell Fucility in Cincinnati Children's Hospital Medical Center.<br>Mouse ES cells, EB3 (RIKEN Bioresource center, AES139), Cells were provided by Dr Hitoshi Niwa in Kumamoto University<br>Mouse ES cells, C57BL/6J-Chr 12A/J/NaJ AC464/GrsJ mES cells (Jackson Laboratory), Cells were provided by Dr Kentaro Iwasawa and Takanori Takebe in Cincinnati Children Hospital Medical Center.
Authentication	H1 cells<br>Cells were authentificated by Pluripotent Stem Cell Fucility in Cincinnati Children's Hospital Medical Center. Cell identity, Genetic stability, and functional pulripotency were checked by STR profiling, G-banded karyotype analysis, and teratoma assay, respectively.<br><br>EB3 cells<br>Cells were authentificated by RIKEN Bioresorce Center (BRC).<br>(http://cellbank.brc.riken.jp/cell_bank/CellInfo/?cellNo=AES0139&lang=Ja#pubEn)<br><br>C57BL/6J-Chr 12A/J/NaJ AC464/GrsJ mES cells<br>Cells were authentificated by The Jackson Laboratory.<br>(https://jackson.jax.org/rs/444-BUH-304/images/004390P8productspec.pdf))
Mycoplasma contamination	H1 cells<br>Mycoplasma comtamination was checked by Pluripotent Stem Cell Fucility in Cincinnati Children's Hospital Medical Center.<br>The result was negative.<br><br>EB3 cells<br>Mycoplasma comtamination was checked by RIKEN BRC and Morimoto's laboratory.<br>The result was negative.<br><br>C57BL/6J-Chr 12A/J/NaJ AC464/GrsJ mES cells<br>Mycoplasma comtamination was checked by Takebe's laboratory.<br>The result was negative.
Commonly misidentified lines<br>(See ICLAC register)	No commonly misidentified cell lines were used in the study.

Animals and other organisms

Policy information about studies involving animals; ARRIVE guidelines recommended for reporting animal research

Laboratory animals	We used C57BL6J mouse for the analyses of wild-type (Figure 2c, 3a, 3e, Supplementary Figure 1, 5, and 6).<br>For the analyses of all mutant mice and littermate control, we used mixed background mice (FIgure 1b-d, 2a, 2b, 2d, 2e, 3b, 3c, Supplementary 2a-c, 3a, 3b, 4, 9).<br>Mice were housed in 18-23 oC with 40-60% humidity. A 12-hour light/12-hour dark cycle was used.<br>We did not distinguish male or female for analyses.
Wild animals	No wild animas were used in the study.
Field-collected samples	No field-collected samples were used in the study.
Ethics oversight	All mouse experiments were approved by the Institutional Animal Care and Use Committee of RIKEN Kobe Branch. Mice were handled in accordance with the ethics guidelines of the institute.

Note that full information on the approval of the study protocol must also be provided in the manuscript.
