# Supplementary material for: Bidirectional Wnt signaling between endoderm and mesoderm confers tracheal identity in mouse and human cells
Source: Nat Commun. 2020 Aug 27;11:4159. doi: 10.1038/s41467-020-17969-w (PMC7453000; doi:10.1038/s41467-020-17969-w)
Supplement: Supplementary file 3 — Supplementary Information [file 41467_2020_17969_MOESM3_ESM.pdf]

Supplementary Information for

Bidirectional Wnt signaling between endoderm and mesoderm confers tracheal identity in mouse and human cells

by Kishimoto et al.

Supplementary Figure 1

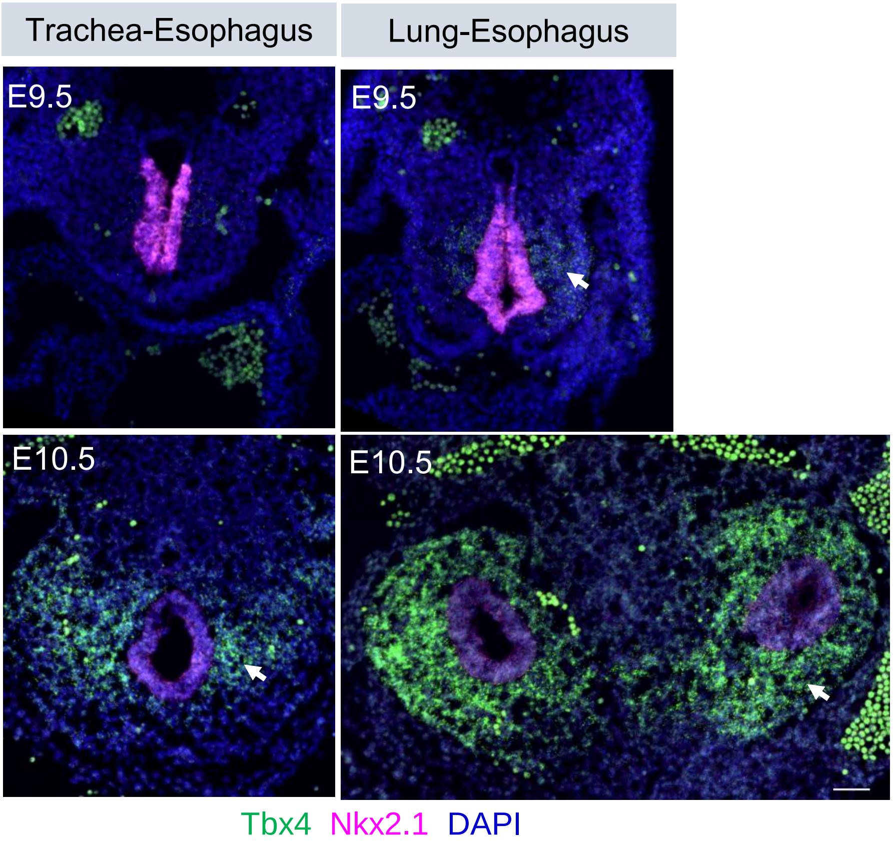

Supplementary Figure 1. *Tbx4* mRNA expression during tracheal-esophageal segregation at E9.5 and 10.5.

RNAscope *in situ* hybridization for *Tbx4* mRNA during tracheoesophageal segregation. Sections were stained by Tbx4 (*green*), Nkx2.1 (*magenta*), and DAPI (*blue*). Arrows indicate $Tbx4^{+}$ cells.

n=2/2 embryos

Scale bar; 50µm

Supplementary Figure 2

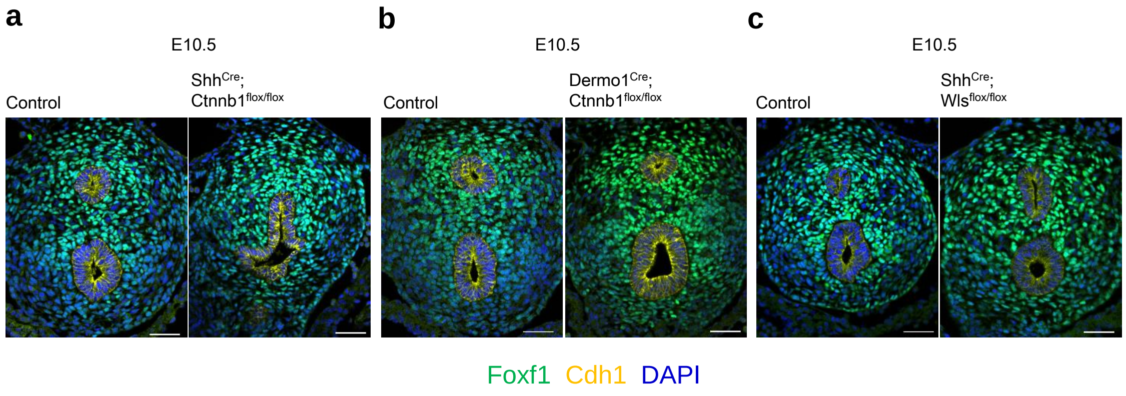

Supplementary Figure 2. Foxf1 expression in *Shh^{Cre}; Ctnnb1$^{flox/flox}$*, *Dermo1^{Cre}; Ctnnb1$^{flox/flox}$*, and *Shh^{Cre}; Wls$^{flox/flox}$* at E10.5.

a, Transverse sections of *Shh^{Cre}*, *Ctnnb1$^{flox/flox}$* mouse embryos and littermate controls. Sections were stained for Foxf1 (*green*), Cdh1 (*yellow*), and DAPI (*blue*). n=2/2 embryos per genotype.

b, Transverse sections of *Dermo1^{Cre}*, *Ctnnb1$^{flox/flox}$* mouse embryos and littermate controls. Sections were stained for Foxf1 (*green*), Cdh1 (*yellow*), and DAPI (*blue*). n=2/2 embryos per genotype.

c, Transverse sections of *Shh^{Cre}*, *Wls$^{flox/flox}$* mouse embryos and littermate controls. Sections were stained for Foxf1 (*green*), Cdh1 (*yellow*), and DAPI (*blue*). n=2/2 embryos per genotype.

Scale bar; 50 μm.

Supplementary Figure 3

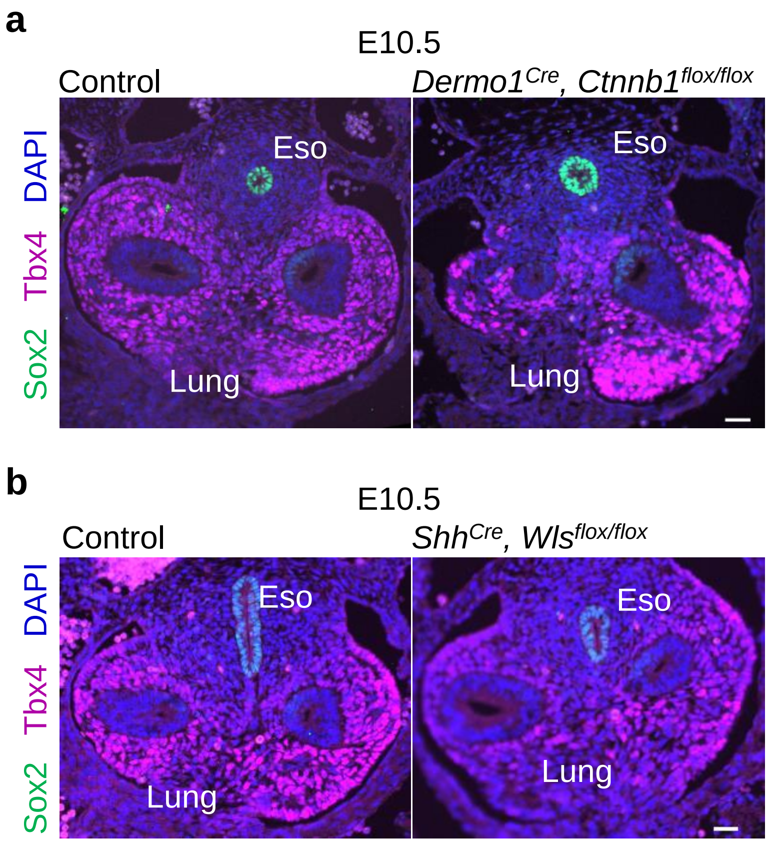

Supplementary Figure 3. Dispensable role of mesodermal Wnt signaling in Tbx4 expression during lung development.

a, Transverse sections of *Dermo1^{Cre}*, *Ctnnb1$^{flox/flox}$* mouse embryos and littermate controls. Sections were stained for Sox2 (*green*), Tbx4 (*magenta*), and DAPI (*blue*). n=3/3 embryos per genotype.

b, Transverse sections of *Shh^{Cre}*, *Wls$^{flox/flox}$* mouse embryos and littermate controls. Sections were stained for Sox2 (*green*), Tbx4 (*magenta*), and DAPI (*blue*). n=3/3 embryos per genotype.

Eso; Oesophagus, Tr; Trachea, Tr-E; Tracheoesophageal tubes

Scale bar; 40 μm.

Supplementary Figure 4

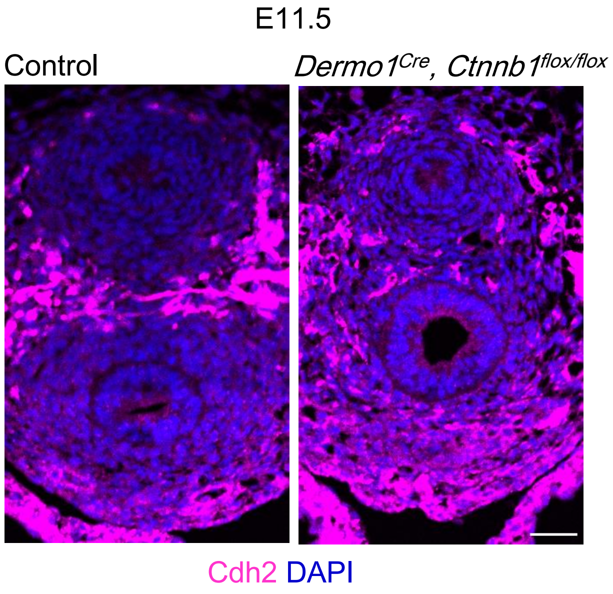

Supplementary Figure 4. Distribution of *Cdh2* in trachea and esophagus in *Dermo1^{Cre}*, *Ctnnb1$^{flox/flox}$* embryos at E11.5

Transverse sections of *Dermo1^{Cre}*, *Ctnnb1$^{flox/flox}$* mouse embryos and littermate controls. Sections were stained for Cdh2 (*magenta*), and DAPI (*blue*). n=2/2 embryos per genotype.

Scale bar; 40µm

Supplementary Figure 5

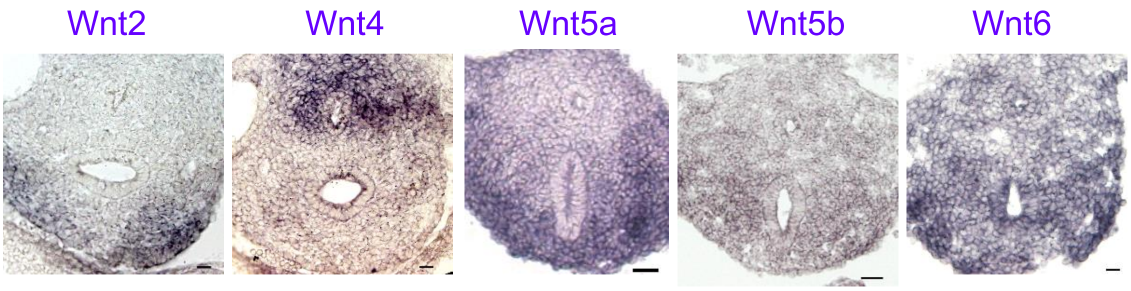

Supplementary Figure 5. Wnt ligand expression in trachea and esophagus at E10.5

In situ hybridization for *Wnt2/4/5a/5b/6* mRNA in mouse embryos at E10.5. n=2/2 embryos per genotype.

Scale bar; 50µm

Supplementary Figure 6

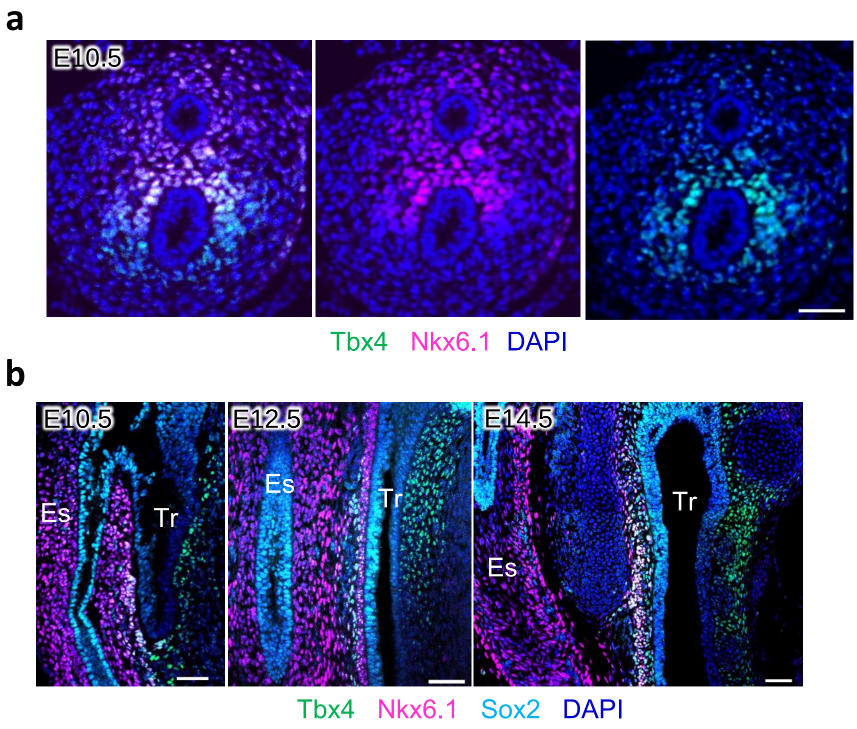

Supplementary Figure 6. *Nkx6.1* and *Tbx4* expression during trachea development.

a, Transverse sections of mouse embryo at E10.5. Sections were stained for Tbx4 (*green*), Nkx6.1 (*magenta*), and DAPI (*blue*). n=2/2 embryos.

b, Sagittal sections of mouse embryo from E10.5 to 14.5. Sections were stained for Tbx4 (*green*), Nkx6.1 (*magenta*), Sox2 (*cyan*) and DAPI (*blue*). n=2/2 embryos at indicated time point.

Es; Esophagus, Tr; Trachea, Scale bar: 50 µm

Supplementary Figure 7

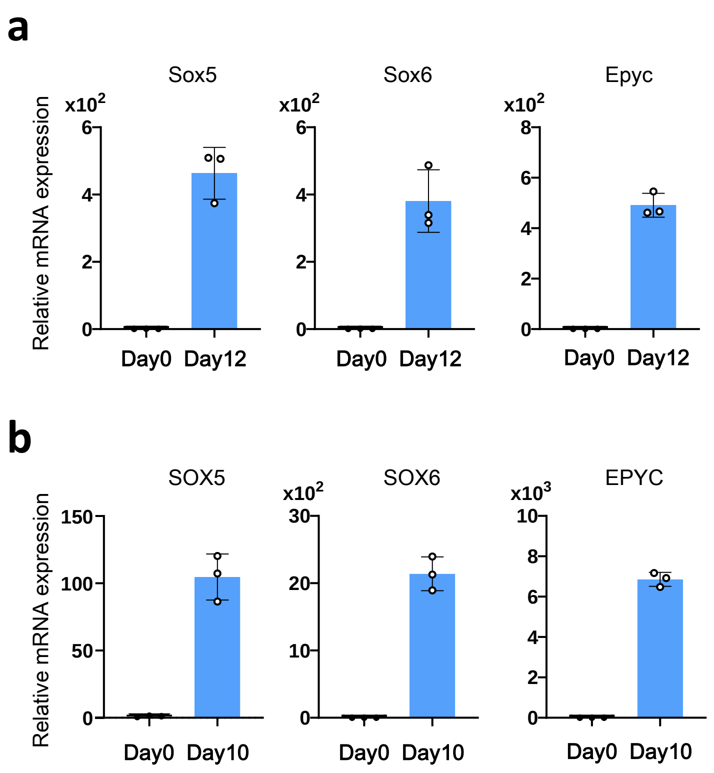

Supplementary Figure 7. Expression of marker genes for chondrocyte differentiation in differentiating cells from mESC and hESC

a, Results of qRT-PCR for *Sox5*, *6* and Epyc expression of mESC-derived tracheal mesodermal cells at day0 and 12 (n=3 independent wells). Experiments were repeated at least twice.

b, Results of qRT-PCR for *SOX5*, *6* and EPYC expression of hESC-derived tracheal mesodermal cells at day0 and 10. (n=3 independent wells). Experiments were repeated at least twice.

Each column shows the mean with S.D.

Source data are provided in Source data file.

Supplementary Figure 8

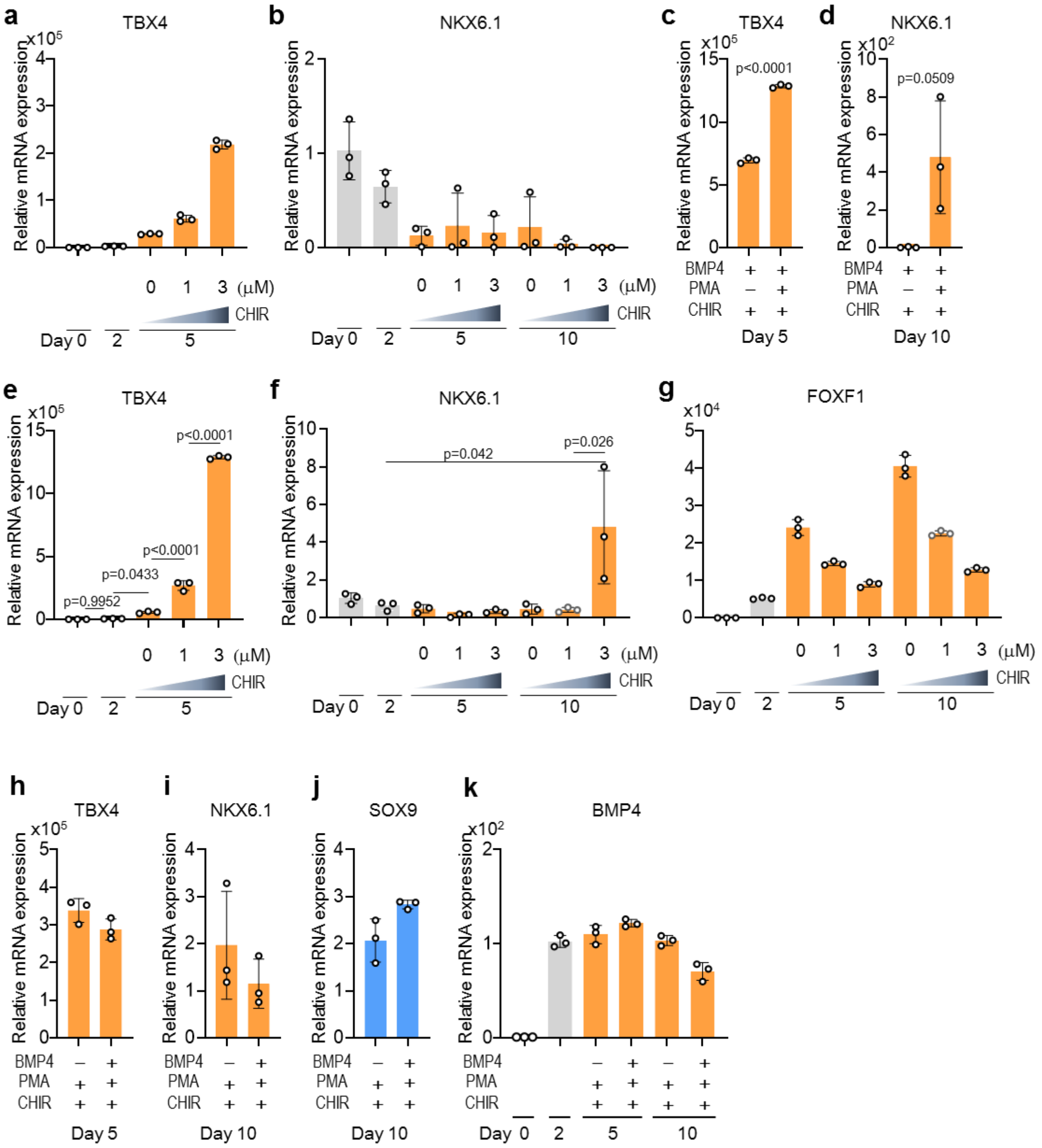

Supplementary Figure 8. Indispensable role of the SHH agonist on tracheal mesoderm differentiation from hESCs.

a and **b**, Results of qRT-PCR for *TBX4* (**a**) and *NKX6.1* (**b**) expression in differentiating hESC-derived cells (n=3 independent wells). Experiments were repeated at least twice. After day2, hLPMs were cultured in cocktails including BMP4 and different doses of CHIR99021.

c and **d,** The results of qRT-PCR for *TBX4* (**c**) and *NKX6.1* (**d**) expression in differentiating hESC-derived cells (n=3 independent wells). Experiments were repeated at least twice. After day2, hLPMs were cultured in cocktails including BMP4 and CHIR99021 with/without purmorphamine (PMA), HH activator.

e-g, qRT-PCR for *TBX4* (**e**), *NKX6.1* (**f**) and *FOXF1* (**g**) expression in differentiating hESC-derived cells (n=3 independent wells). Experiments were repeated at least twice. After day2, hLPMs were cultured in cocktails including PMA, BMP4 and different doses of CHIR99021.

h-j, qRT-PCR of *TBX4* (**e**), *NKX6.1* (**f**), *SOX9* (**g**) in differentiating hESC-derived cells (n=3 independent wells). Experiments were repeated at least twice.After day2, hLPMs were cultured in PMA and CHIR99021 with/without BMP4.

k, qRT-PCR for *BMP4* expression in differentiating hESC-derived cells at different time points (n=3 independent wells). Experiments were repeated at least twice. After day2, hLPMs were cultured in PMA and CHIR99021 with/without BMP4.

Each column shows the mean with S.D. P-values were provided by two-sided Student t-test (**c, d**) or two-sided Tukey's multiple comparison (**e**, **f**). *p<0.05, **p<0.005, ***p<0.0001

Source data are provided in Source data file.

Supplementary Figure 9

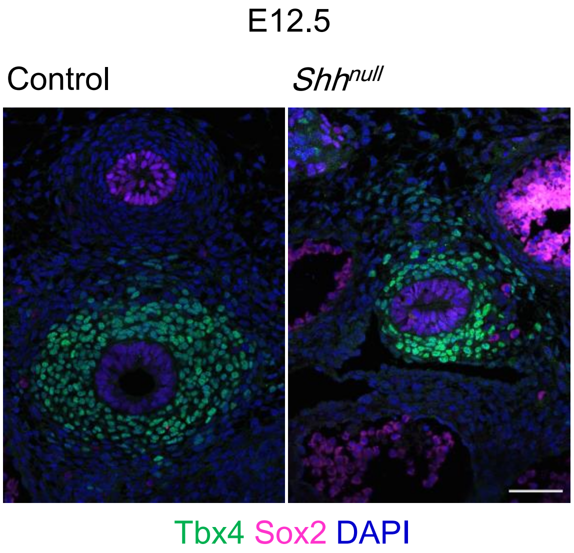

Supplementary Figure 9. Tbx4 expression in Shh^{null} embryos at E12.5

Transverse sections of Shh^{null} embryo and littermates control at E12.5. Sections were stained for Tbx4 (*green*), Sox2 (*magenta*), and DAPI (*blue*). n=2/2 embryos per genotype. Scale bar; 50μm

Supplementary Table 1 Methods for Immunohistochemistory

Antibody (Dilution)	*Company, Catalog code*	*Fixative*	*Tissue preparation*	*Antigen retrieval*	*Secondary antibody*
□-Smooth muscle actin(SMA)-Cy3 (1:1000)	Sigma, C6198	4% PFA overnight at 4˚C	Paraffin	Histo^{VT} One 15min at 105˚C	-
Cdh1 (1:500)	Cell Signaling Technology #3195	4% PFA overnight at 4˚C	Paraffin	Histo^{VT} One 15min at 105˚C	Rabbit Alexa594
Foxf1 (1:500)	R&D systems, AF4798	4% PFA overnight at 4˚C	Paraffin	Histo^{VT} One 15min at 105˚C	Goat Alexa488
GFP (1:200)	Thermo Fisher Scientific, A10262	4% PFA overnight at 4˚C	Paraffin	Histo^{VT} One 15min at 105˚C	Chicken Alexa488
Nkx2.1 (1:400)	Santa Cruz Biotechnology, Inc, sc-13040	4% PFA overnight at 4˚C	Paraffin	Histo^{VT} One 15min at 105˚C	Rabbit Alexa555
Nkx6.1 (1:100)	Developmental Studies Hybridoma Bank, F55A12	4% PFA overnight at 4˚C	Paraffin	Histo^{VT} One 15min at 105˚C	Mouse Alexa647
Sox2 (1:400)	Santa Cruz Biotechnology, Inc, sc-17320	4% PFA overnight at 4˚C	Paraffin	Histo^{VT} One 15min at 105˚C	Goat Alexa488
Sox9 (1:1000)	Abcam, AB5535	4% PFA overnight at 4˚C	Paraffin	Histo^{VT} One 15min at 105˚C	Rabbit Alexa488
Tbx4 (1:300)	Abcam, ab220035	4% PFA overnight at 4˚C	Paraffin	Histo^{VT} One 15min at 105˚C	Rabbit Alexa594

Supplementary Table 2 Primer lists for the construction of *in situ* hybridization probes

Probe	*Primer sequence (Forward, 5' to 3')*	*Primer sequence (Reverse, 5' to 3')*	*Accession number*	*Nucleotides*
Wnt2	ATAGTCGACACAGAGATCACAGCCTCTTT	ATAGAATTCCATGTCCTCAGAGTACAGGA	NM_023653	429-817
Wnt4	ATAGTCGACAGAAACTCAAAGGCCTGATC	ATAGAATTCTCGTTGTTGTGAAGATTCATGA	NM_009523	176-67
Wnt5a	ATAGTCGACATGGCTTTGGCCACGTTT TT	ATAGAATTCATTTGCATCACCCTGCCAAA	NM_001256224	95-402
Wnt7b	ATAGTCGACTGAACCTTCACAACAATGAGG	ATAGAATTCAGTGGAATTTGCAGTTACACT	NM_001163633	947-1396

Supplementary Table 3 Methods for RNAscope experiments

Probe	*Catalog code*	*Fixative*	*Tissue preparation*	*Target Retrieval*	*Protease*	*Fluorophore*
Axin2	400331-C2	4% PFA overnight at 4˚C	Frozen	1xTarget retrieval 5min at 98-102˚C	Protease III 15min at 40 ˚C	Opal520
Nkx2.1	434728-C3	4% PFA overnight at 4˚C	Frozen	1xTarget retrieval 5min at 98-102˚C	Protease III 15min at 40 ˚C	Opal570
Tbx4	483291	4% PFA overnight at 4˚C	Frozen	1xTarget retrieval 5min at 98-102˚C	Protease III 15min at 40 ˚C	Opal690

Supplementary Table 4 Methods for Immunocytochemistory

Antibody (Dilution)	*Company, Catalog code*	*Fixative*	*Secondary antibody*
Aggrecan (1:100)	Abcam, ab3778	4% PFA 30 min at RT	Mouse Alexa647
□-SMA (1:200)	Sigma, #A2547	4% PFA 30 min at RT	Mouse Alexa647
□-SMA-Cy3 (1:1000)	Sigma, C6198	4% PFA 30 min at RT	-
Collagen1a1 (1:200)	Abcam. ab34710	4% PFA 30 min at RT	Rabbit Alexa546
Collagen2a1 (1:200)	Santa Cruz Biotechnology, Inc, sc-7764	4% PFA 30 min at RT	Goat Alexa488
Foxf1 (1:500)	R&D systems, AF4798	4% PFA 30 min at RT	Goat Alexa488
Gata4 (1:200)	Santa Cruz Biotechnology, Inc, sc-1237	4% PFA 30 min at RT	Goat Alexa488
Nkx6.1 (1::100)	R&D systems, AF5857	4% PFA 30 min at RT	Goat Alexa594
Sox9 (1:1000)	Abcam, AB5535	4% PFA 30 min at RT	Rabbit Alexa488
Tagln (1:200)	Abcam, ab14106	4% PFA 30 min at RT	Rabbit Alexa 594

Supplementary Table 5 Primer lists for quantitative RT-PCR (Mouse)

	Primer sequence (Forward, 5' to 3')	*Primer sequence (Reverse, 5' to 3')*
Acta2	ACTGGGACGACATGGAAAAG	GTTCAGTGGTGCCTCTGTCA
Bmp4	GCCGAGCCAACACTGTGAGGA	GATGCTGCTGAGGTTGAAGAGG
Epyc	TTCTGGGTCCACACACCAAC	CTTCTTGGGCAGTGGAGGAATA
Foxf1	CCTGTCTGGCAGCATCTCCAC	GACTGTGAGTGATACCGAGGGA
Gapdh	TCACCACCATGGAGAAGGC	GCTAAGCAGTTGGTGGTGCA
Gata4	GCCTCTATCACAAGATGAACGGC	TACAGGCTCACCCTCGGCATTA
Hoxb6	GCTCTACTCGTCTGGCTATGC	GTGGGTAATAGGAGGACGCC
Prrx1	GACACCCCTCAGCAGGACAA	TGAAACCACACCTGCACTCT
Sox5	ACATGCACAATTCCAACATCAGC	GGTCATAGCTTTCCAGCGAGAT
Sox6	AATGCACAACAAACCTCACTCT	AGGTAGACGTATTTCGGAAGGA
Sox9	TGAGAGGTTTCAGATGCAGTG	CACATCCACATACAGTCCAGG
Tbx4	TCACTGGATGCGGCAGTTGGTCTCT	CACGTGGGTGCAAAAGGCTGTGTTT
Tbx5	GGACCCAGTCCCTTGAATGG	TCCAGGCTGAGGAGTTCTAGGC
Wnt2	CCAACGAAAAATGACCTCGT	GGGAAGTCAAGTTGCACACA

Supplementary Table 6 Primer lists for quantitative RT-PCR (Human)

	Primer sequence (Forward, 5' to 3')	*Primer sequence (Reverse, 5' to 3')*
ACTA2	CTATGCCTCTGGACGCACAACT	CAGATCCAGACGCATGATGGCA
BMP4	CAAACTTGCTGGAAAGGCTC	CCGCTACTGCAGGGACCTAT
EPYC	AGGAGGAGGAATCTACTCCCA	CAGCGGAGGAATAGCATCAAG
FOXF1	AGCAGCCGTATCTGCACCAGAA	CTCCTTTCGGTCACACATGCTG
GAPDH	CCCATCACCATCTTCCAGGAG	CTTCTCCATGGTGGTGAAGACG
GATA4	TAGCCCCACAGTTGACACAC	GTCCTGCACAGCCTGCC
HOXB6	CACTCCGGTCTACCCGTGGATGCA	CATATCTTGATCTGCCTCTCCGTCAG
NKX6.1	ATGACAGAGAGTCAGGTCAAGG	CTCCGAGTCCTGCTTCTTCTT
PRRX1	TGCAGGCTTTGGAGCGTGTCTT	CTCATTCCTGCGGAACTTGGCT
SOX5	CAGCCAGAGTTAGCACAATAGG	CTGTTGTTCCCGTCGGAGTT
SOX6	TTACTCGGCCAGAAGATGCAG	ACTCGTGCTTCAGCCACAGT
SOX9	GTAATCCGGGTGGTCCTTCT	GTACCCGCACTTGCACAAC
TBX4	TGATCATCACTAAGGCTGGCAG	ACAGAACTTGTAGCGATGGTCAT
TBX5	ACAAAGTGAAGGTGACGGGCCTTA	ATCTGTGATCGTCGGCAGGTACAA
WNT2	CTGTATCAGGGACCGAGAGG	CCCACAGCACATGACTTCAC
